# Supplementary figures and images for: Dysregulated H19/Igf2 expression disrupts cardiac-placental axis during development of Silver-Russell syndrome-like mouse models
Source: eLife. 2022 Nov 28;11:e78754. doi: 10.7554/eLife.78754 (PMC9704805; doi:10.7554/eLife.78754)

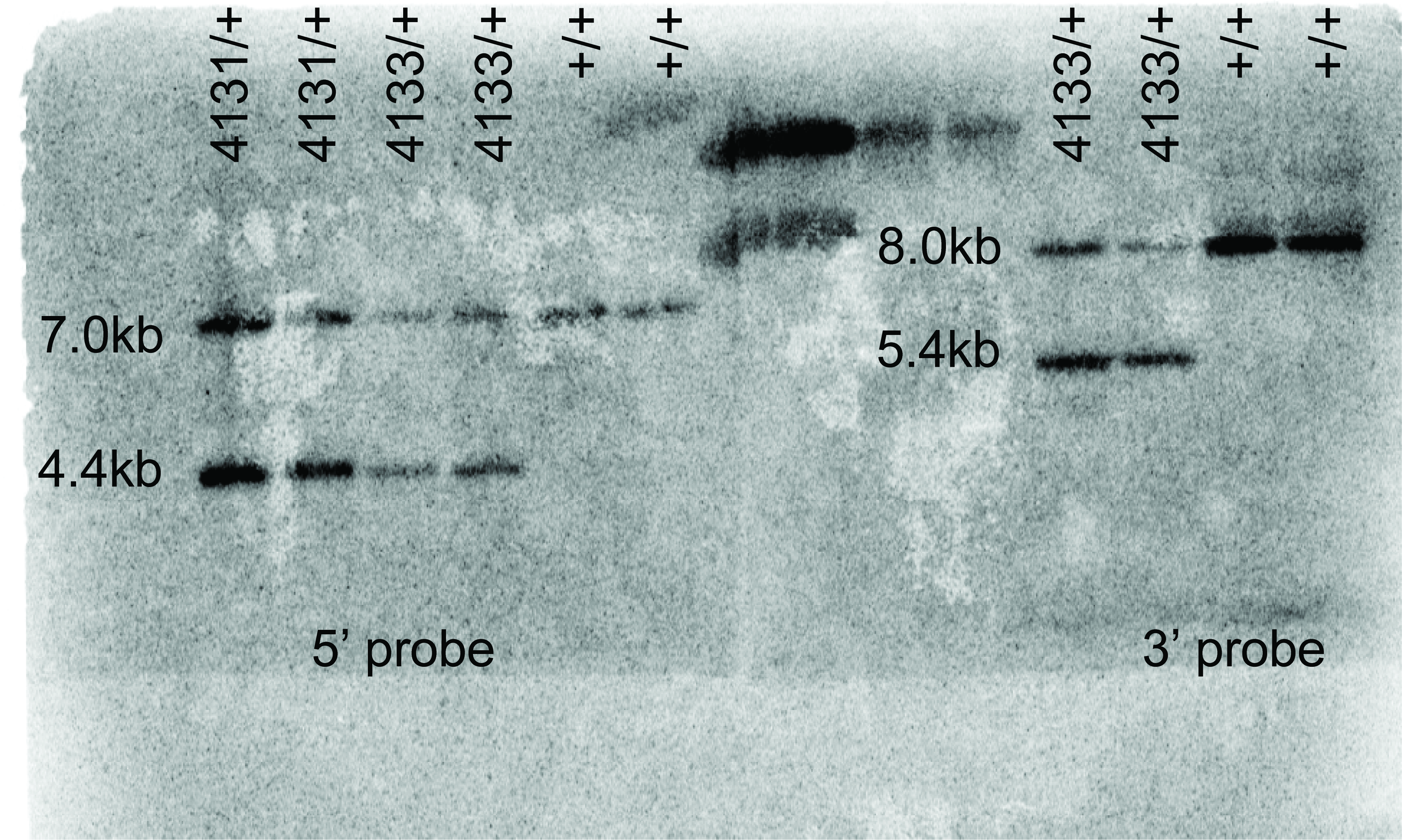

Supplement: Figure 4—figure supplement 1—source data 1. [file elife-78754-fig4-figsupp1-data1.zip › Figure Supplement 3B Source data/Supplemental Figure 3B - source data 2_southern blot_labelled.tif]

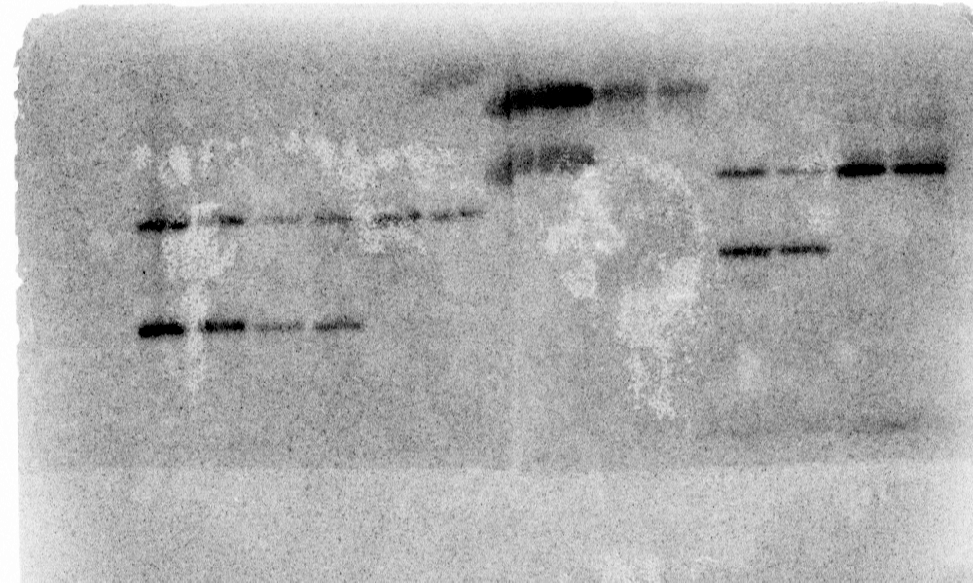

Supplement: Figure 4—figure supplement 1—source data 1. [file elife-78754-fig4-figsupp1-data1.zip › Figure Supplement 3B Source data/Supplemental Figure 3B - source data 1_southern blot_raw.bmp]

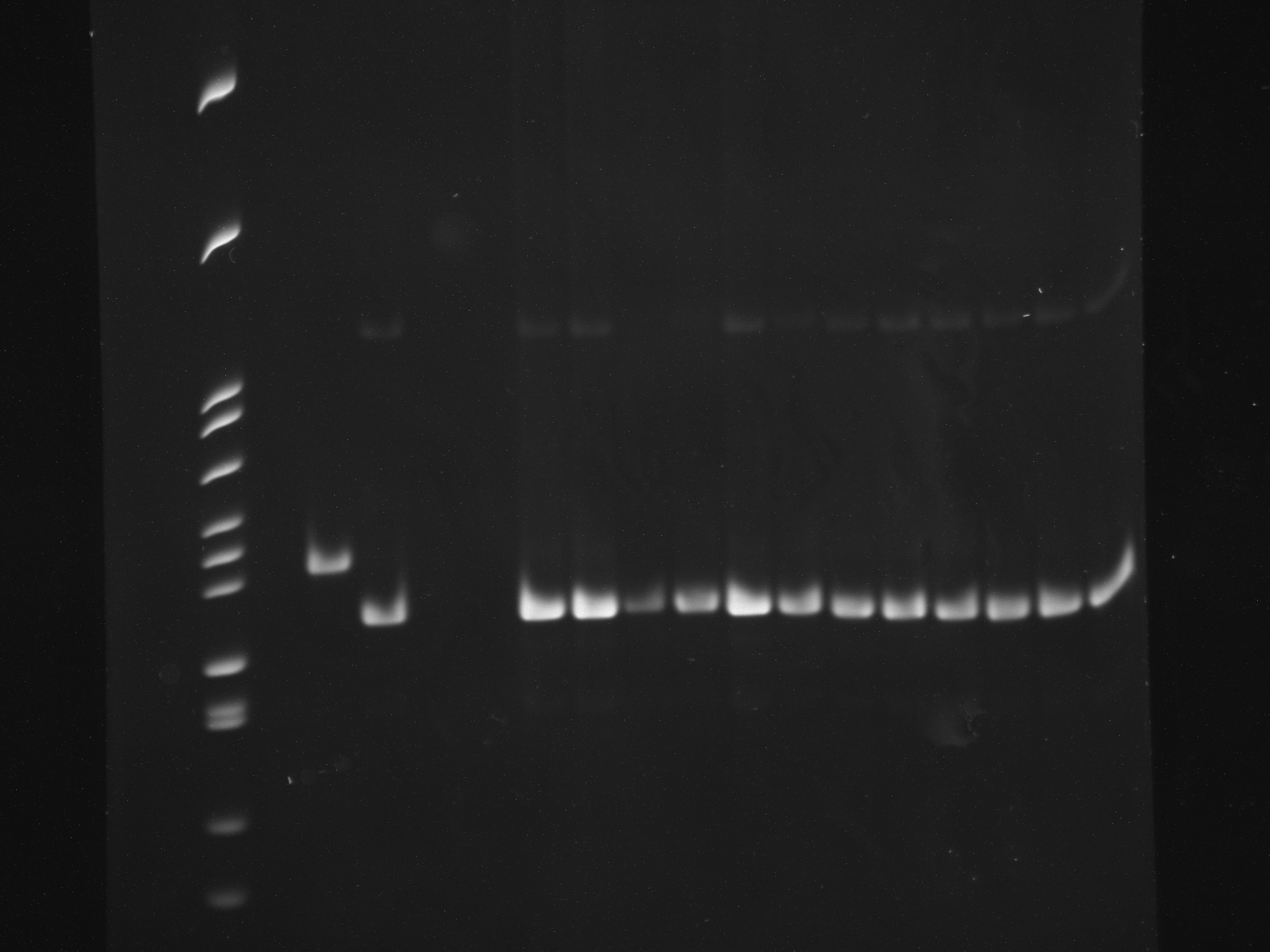

Supplement: Figure 4—figure supplement 1—source data 3. [file elife-78754-fig4-figsupp1-data3.zip › Figure Supplement 3C Source data_raw gels/Supplemental Figure 3C - source data 2_Liver_raw.tif]

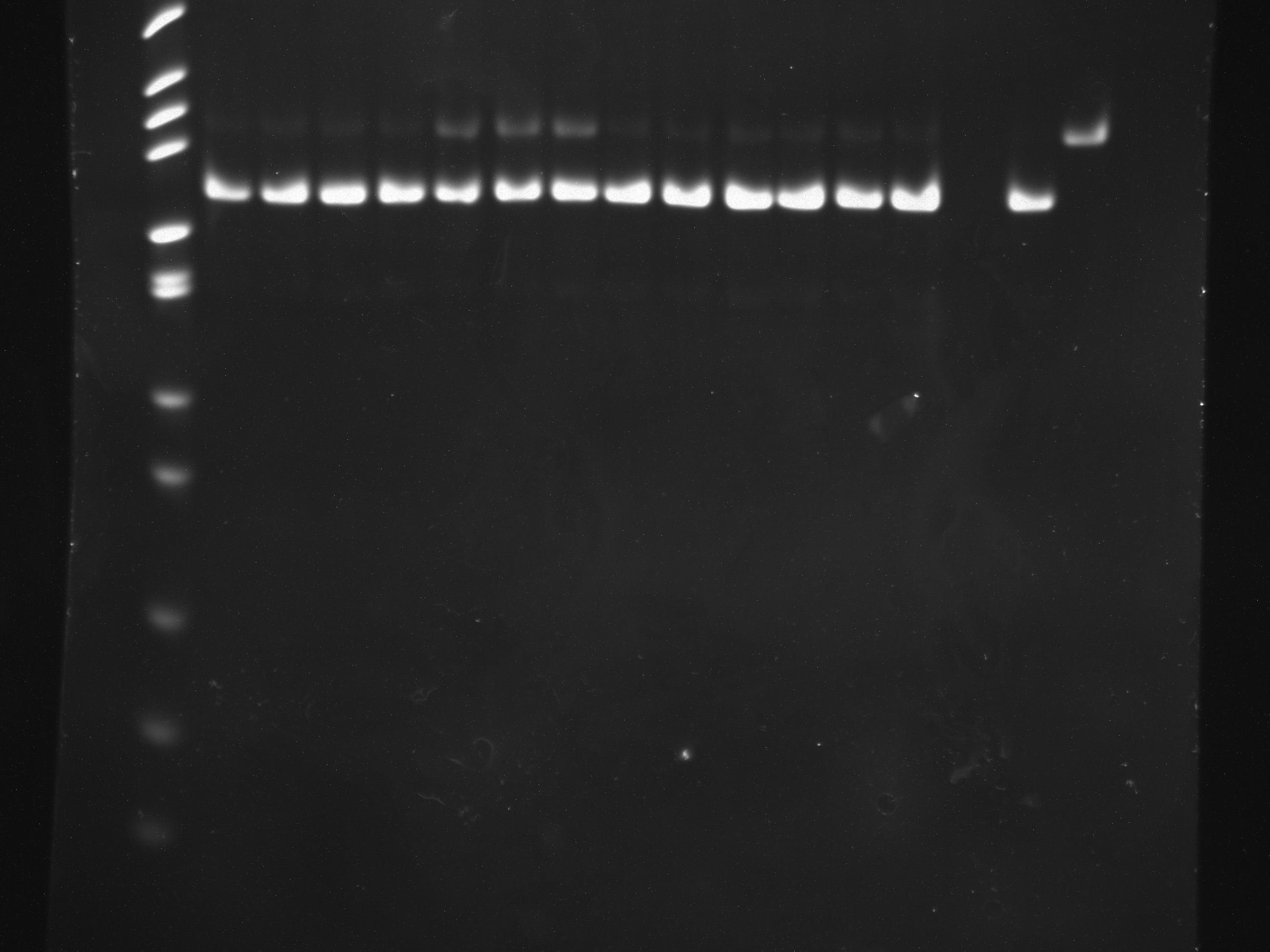

Supplement: Figure 4—figure supplement 1—source data 3. [file elife-78754-fig4-figsupp1-data3.zip › Figure Supplement 3C Source data_raw gels/Supplemental Figure 3C - source data 1_Tongue_raw.tif]

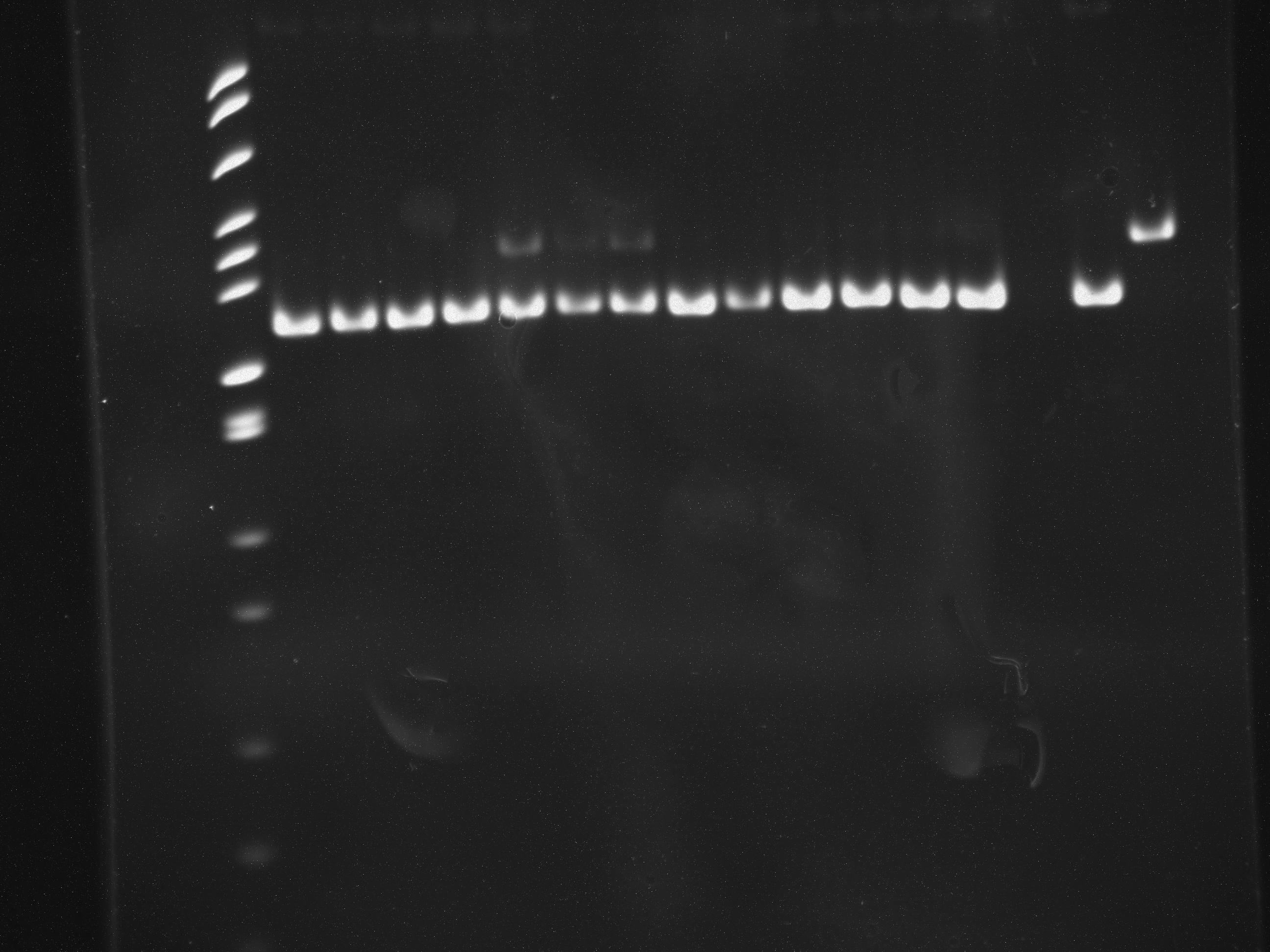

Supplement: Figure 4—figure supplement 1—source data 3. [file elife-78754-fig4-figsupp1-data3.zip › Figure Supplement 3C Source data_raw gels/Supplemental Figure 3C - source data 3_Heart_raw.tif]
