# Supplementary material for: Dysregulated H19/Igf2 expression disrupts cardiac-placental axis during development of Silver-Russell syndrome-like mouse models
Source: eLife. 2022 Nov 28;11:e78754. doi: 10.7554/eLife.78754 (PMC9704805; doi:10.7554/eLife.78754)
Supplement: Supplementary file 1. [file elife-78754-supp1.docx]

Supplementary File 1. Primers and PCR conditions utilized in this study.

| **Primer** | **Forward** | **Reverse** | **Reference** |
| --- | --- | --- | --- |
| **Mouse qRT-PCR primers** | | | |
| *Arbp* | TCCCACTTACTGAAAAGGTCAAG | TCCGACTCTTCCTTTGCTTC | (Hur et al., 2016) |
| *Nono* | GCTCGTGAGAAGCTGGAGAT | TTCTTGACGTCTCATCAAATCC | (Plasschaert & Bartolomei, 2014) |
| *Rpl13a* | ATCCCTCCACCCTATGACAA | GCCCCAGGTAAGCAAACTT |  |
| *H19* | GTCTCGAAGAGCTCGGACTG | ACTGGCAGGCACATCCAC | (Hur et al., 2016) |
| *Igf2* | CGCTTCAGTTTGTCTGTTCG | GCAGCACTCTTCCACGATG | (Weaver et al., 2009) |
| **Mouse allele-specific expression primers** | | | |
| *Igf2* | ATCTGTGACCTCTTGAGCAGG | GGGTTGTTTAGAGCCAATCAA | (de Waal et al., 2015) |
| PCR condition: 95°C 2min, (95°C 15s, 58°C 10s, 72°C 20s)x26-30 cycles, 72°C 5min | | | |
| **Mouse genotyping primers** | | | |
| *hIC1* | CCTTCACGGCTTTGACACTC | GTCAACCGGAGGCACAGTAT | (Hur et al., 2016) |
| *△H19* | TTGTGGTGAGGCTGTCTTTG | CCTATTCCCCATTCCATCCT | This study |
| *△3.8* | CCAACTGAGAGGGCCATAGTGTGAG | CCACAGAGTCAGCATCCAC | (Thorvaldsen et al., 2002) |
| PCR condition: 95°C 2min, (95°C 15s, 58°C 10s, 72°C 20s)x35 cycles, 72°C 2min | | | |
| **gRNA pairs for generating *△H19* allele** (PAM sequences are underlined) | | | |
| Pair A | CTTCAATATAATGCGACTCATGG | AACGTGCGCTGGAACGATACAGG | |
| Pair B | CAATATAATGCGACTCATGGGGG | ATCAGTACATGGCCCCGCCGGGG | |
| Primers for px335 plasmid amplification | TTAATACGACTCACTATAGGNNNNNNNNNNNNNNNNNNNNGTTTTAGAGCTAGAAATAGC (The underlined nucleotides were substituted with each gRNA sequence excluding the PAM sequence) | AGCACCGACTCGGTGCCACT | |
